# Collective sensing and collective responses in quorum-sensing bacteria

**DOI:** 10.1098/rsif.2014.0882

**Published:** 2015-02-06

**Authors:** R. Popat, D. M. Cornforth, L. McNally, S. P. Brown

**Affiliations:** 1 Centre for Immunity, Infection and Evolution, School of Biological Sciences, University of Edinburgh, Edinburgh EH9 3JT, UK; 2 Molecular Biosciences, University of Texas at Austin, 2500 Speedway NMS 3.254, Austin, TX 78712, USA

**Keywords:** quorum sensing, collective behaviour, systems biology, social evolution

## Abstract

Bacteria often face fluctuating environments, and in response many species have evolved complex decision-making mechanisms to match their behaviour to the prevailing conditions. Some environmental cues provide direct and reliable information (such as nutrient concentrations) and can be responded to individually. Other environmental parameters are harder to infer and require a collective mechanism of sensing. In addition, some environmental challenges are best faced by a group of cells rather than an individual. In this review, we discuss how bacteria sense and overcome environmental challenges as a group using collective mechanisms of sensing, known as ‘quorum sensing’ (QS). QS is characterized by the release and detection of small molecules, potentially allowing individuals to infer environmental parameters such as density and mass transfer. While a great deal of the molecular mechanisms of QS have been described, there is still controversy over its functional role. We discuss what QS senses and how, what it controls and why, and how social dilemmas shape its evolution. Finally, there is a growing focus on the use of QS inhibitors as antibacterial chemotherapy. We discuss the claim that such a strategy could overcome the evolution of resistance. By linking existing theoretical approaches to data, we hope this review will spur greater collaboration between experimental and theoretical researchers.

## 1. Introduction

**Figure 1.**
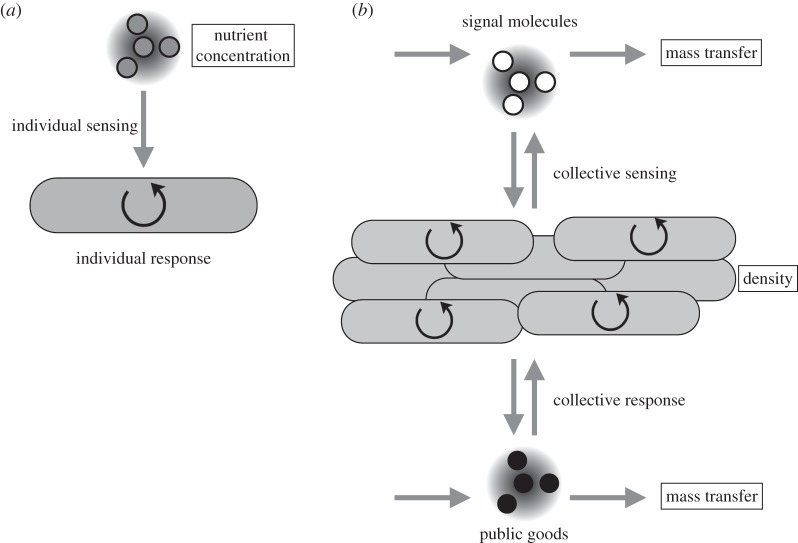
Individual sensing versus collective sensing and responses. (*a*) An individually sensed environmental parameter such as lactose concentration is sensed by an individual cell and affects an individual response. The *lac* operon is upregulated, and lactose transport and metabolism is enhanced. Such a decision can be made by directly sensing the nutrient concentration and an effective response is not contingent upon the action of others. (*b*) By contrast, environmental parameters that cannot be directly sensed such as population density and mass transfer can affect the concentration of QS molecules. Multiple individuals contribute to a common pool of molecular environmental probes generating information at the group level, via a collective mechanism of sensing. The resulting change in behaviour involves both individual traits and group traits (in particular, secretions) that favourably modify the environment.

Bacteria are prodigious decision-makers, responding to multiple abiotic and biotic environmental challenges with changes in gene expression [1]. The extent of investment in decision-making varies across bacterial species but is often impressive, with gene regulatory elements comprising between 1 and 10% of the genome [2,3]. For instance, the classic bacterial decision-making mechanism, the *lac* operon, controls whether *Escherichia coli* cells invest in the metabolism of lactose, as a function of its availability in the environment [4]. The regulation of the *lac* operon and lactose metabolism links *directly sensed* environmental information (nutrient concentrations) to an *individually orchestrated* response (catabolic pathway expression). Such decision-making phenomena can therefore be studied at the level of the individual bacterial cell and the intracellular molecular network underlying the decision-making process (figure 1*a*).

Over the past few decades, it has become increasingly clear that bacterial decision-making routinely exceeds the two limits of (i) individual sensing and (ii) individual responses exemplified by the *lac* operon. In addition to individually sensing directly assessable environmental properties such as nutrient concentrations or temperature, many bacterial species engage in indirect mechanisms of environment sensing, via emission and detection of diffusible small molecules, in a process known as ‘quorum sensing’ (QS) [5–7]. The information provided by the extracellular titre of signal molecules then shapes large-scale changes in gene expression, controlling both intracellular (individual) and extracellular (collective) responses (figure 1*b*).

Behaviour under the management of QS includes spectacular feats of group activity, including social motility (swarming), colony luminescence, biofilm formation and extracellular digestion [8]. QS also controls a range of individual traits, including specific nutrient catabolism [9] and genetic competence [10]. In this review, we examine the collective nature of both the harvesting of information by QS mechanisms, and of the response to QS signal inputs. We discuss what information is harvested by QS, and why QS preferentially controls collective and coordinated responses. We review the evolutionary dynamics of QS in particular in the context of social conflict in genetically mixed groups. Finally, we comment on the growing interest in using QS as a novel target for antibacterial chemotherapy, highlighting the potential evolutionary responses to ‘QS-interference’ drugs and the possibility of ‘evolution proof’ treatments.

We refer often to examples from the environmental generalist and opportunistic pathogen *Pseudomonas aeruginosa*, because it is a model organism for social behaviour and communication in microbes [11–16]. However, the ideas are broadly applicable and the organism shares fundamental similarities with many other QS bacteria. This is not an exhaustive review of QS, our focus is on understanding the population-scale properties of QS and we refer the reviewer to several excellent papers on the intracellular properties of QS, both theoretical and empirical [7,17–19].

## 2. What does quorum-sensing sense?

While the mechanistic underpinnings of QS have been described for many species in exquisite detail, the functional significance of QS is still disputed, with several hypotheses competing to explain how QS contributes to bacterial fitness [20–22]. The classical view is that QS allows bacteria to sense and respond appropriately to different levels of bacterial population density. It is clear that all else being equal, more cells in a defined space will lead to higher concentrations of signal molecule, allowing the signal molecule to serve as a proxy for cell density [23]. The main alternative ‘diffusion sensing’ (DS) hypothesis [20] argues that variation in the concentration of extracellular signal molecules will primarily be shaped by physical mass transfer forces such as diffusion or advection, rather than bacterial density. Redfield [20] argues that the focus on density has been spurred by undue attention to the artificial growth conditions in most laboratory work; the high-density, clonal growth of a single lineage in large volumes of sterile rich media is very different from bacterial growth in natural populations. Outside the laboratory, bacterial growth is typically constrained to far lower densities, and so she argues the primary information encoded by variation in signal concentration is variation in the mass transfer properties of the local environment. For example, QS molecules are more likely to accumulate in viscous environments where their rate of removal is reduced [24]. Cells are then able to use QS molecules as cheap environmental probes and limit the production of costly secreted products such as exoenzymes to when they will remain nearby.

**Figure 2.**
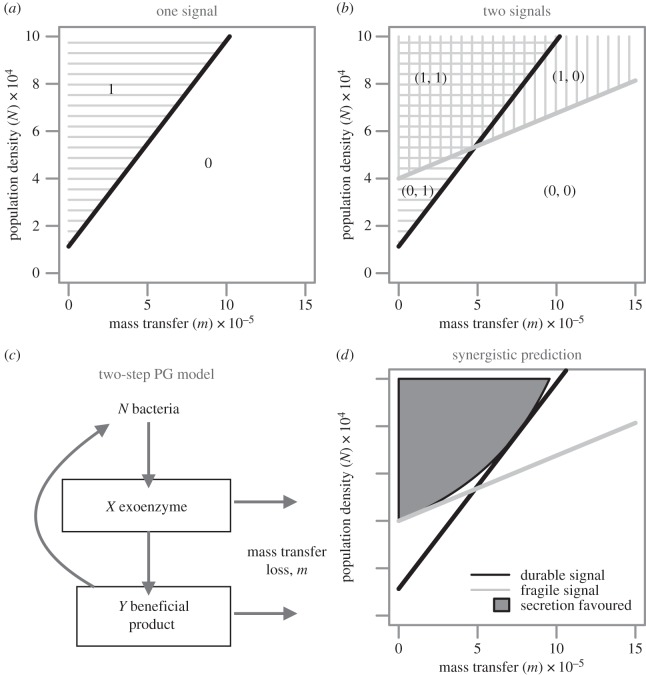
Signal ambiguity and multiple signals. (*a*) The ambiguity between population density and mass transfer is inherent when inferences are made on the concentration of only one QS molecule. (*b*) With two molecules that have differing rates of chemical decay, there are non-overlapping regions in their thresholds over population density and mass transfer allowing greater environmental resolution (see box 1), requiring combinatorial responses to the concentration of the two molecules. (*c*) A two-step public goods model where a beneficial secreted product liberates nutrients in the environment. Both the secreted product and the liberated nutrient can be lost via mass transfer (see box 2). (*d*) Secretions are more effective at high concentrations and therefore at high population density and low mass transfer. The benefit derived from secretions that liberate nutrients from the environment is affected by both the loss of the secretion and the liberated nutrient (see box 2). This double jeopardy contributes to an accelerating penalty on the benefit of secretion with increasing mass transfer which translates into the curved grey shaded region in panel *c* (the region favouring investments in secreted public goods). This region can be better approximated by two signals and an AND-gate response rule. The thick lines represent the threshold beyond which QS is ‘on’ (1) and below which QS is ‘off’ (0). The dark grey region in (*c*) represents the mass transport and population density regimes where secretions that liberate nutrients would be favoured. Parameters for the two signal molecules are: *u*_1_ = 1.3 × 10^−5^ s^−1^, *a*_1_ = 1.15 × 10^−9^ cell s^−1^, *u*_2_ = 1.45 × 10^−4^ s^−1^, *a*_2_ = 3.625 × 10^−9^ cell s^−1^. The parameters for the public good model in panel *c* are: *P* = 9.6 × 10^−9^ µg ml s^−1^, *q* = 10^−1^ s^−1^, *e* = 4 × 10^−3^ s^−1^, *f* = 1.2 × 10^−3^ s^−1^, *c* = 7 × 10^−7^ µl cell s^−1^. See box 1 for model details. Adapted from [25].

The ‘DS’ argument and the classical QS argument stand in conflict because neither can be true in their purest form. If cells attempt to infer their density by sensing QS molecule concentration, their inferences would be confounded by variation in the mass transfer properties of the environment and vice versa. Box 1 and figure 2*a* illustrate the basic argument schematically. With one signal molecule and a predictable mass transfer regime (i.e. a known value of mass transfer rate *m*), signal concentration is informative of whether bacterial density is above or below a threshold value of *N* (i.e. classic QS). However, it is also true that for the same signal molecule and a predictable density (i.e. a known value of *N*), signal concentration is also informative of the mass transfer regime (DS). For bacteria experiencing uncertainty over both *N* and *m*, estimates of either parameter are confounded by uncertainty over the other, so that high-density, high-mass transfer environments can be indistinguishable from low-density, low mass transfer environments.

Box 1. **Dynamics of extracellular signal concentrations.**

We will consider very simple models for the extracellular dynamics of signal molecule concentrations, taken from Cornforth *et al*. [25]. In our models, signal molecules are lost by two factors: decay of the molecules themselves at rates specific to each secreted molecule, and mass transfer (specifically, advection). In our model of signal density, the local density of signal (*S*) is increased by the production (at baseline *per capita* rate *p*) of signal by local bacteria (at density *N*) and is decreased by mass transfer (at rate *m*; independent of molecular design) and by physical decay (at rate *u*; sensitive to molecular design). Autoinduction is represented by *aS*, which is the rate of increased signal induction dependent on present signal concentration. Note that we can conveniently assume that bacterial density *N* is static, as *P. aeruginosa* only responds to signal when growth is limited [26]. In short, QS is used as a device to diagnose and overcome road-blocks preventing further growth. The dynamics of two distinct signal molecules is given by the equations:
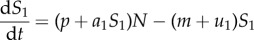
and
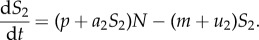

For each, the equilibrium is given by S*_k_ = *Np*/(*m* − *a_k_N* + *u_k_*). At sufficiently low-density and/or high-mass transfer regimes, the equilibrium is stable (when *Na_k_* < *m* + *u_k_*), and we consider the autoinduction process to be ‘off’. By contrast, when *Na_k_* > *m* + *u_k_* , the equilibrium becomes unstable (leading to an unconfined increase in *S_k_*), and we consider autoinduction to be ‘on’ (figure 2*a*,*b*).

A simple model describing the dynamics of extracellular signal molecule concentration. The model highlights that the ambiguity between different environmental axes of variation can be resolved by using multiple signals and combinatorial response rules (see figure 2*a*,*b*).

A possible resolution to the social environment sensing versus physical environment sensing debate is suggested by Hense *et al*. [21]. They argue that while signal molecules can accumulate due to high population density and/or low removal rate, the appropriate response in each case is the same: upregulate secreted factors to exert control over the extracellular environment. This ‘efficiency-sensing’ hypothesis posits that the signal molecules serve as cheap test-cases for extracellular investment; if signal concentration is high then this implies that more costly secreted enzymes will also achieve high concentrations, owing to a favourable combination of limited mass transfer and/or complementation from neighbouring cell production. Consistent with this, many traits controlled by QS are secretions [27,28]. However, the logic of efficiency sensing alone does not account for non-secreted traits under QS control, for example, the control of luminescence via QS in *Vibrio fischeri*—the canonical QS-mediated trait [29]. The effectiveness of group luminescence is certainly coupled to density but is not directly affected by the mass transfer properties of the environment. Mass transfer, however, can still have a substantial impact on QS molecule concentration. Most generally of all, the molecular properties and therefore the dynamics of QS signal molecules are likely to differ significantly from that of the effector proteins and molecules released in response. This means the extracellular fate of QS molecules may not be predictive of the extracellular fate of response proteins such as enzymes. In addition to this, most secretions do not provide benefits directly, but instead confer benefits on cells by modifying their environment (e.g. digestion of substrates by exoenzymes). As the final products of secretions are likely to also be subject to mass transfer, efficiency-sensing can fail in these scenarios (see box 2 and figure 2*d*).

Box 2. **Two-stage public goods model.**

Consider a secreted exoproduct of concentration *X* that interacts with the environment to release a beneficial shared nutrient *Y* (figure 2*c*). For instance, secreted iron scavenging siderophore molecules bind to iron and can then be imported by bacteria, and secreted protease enzymes break down a protein into usable amino acids. This ‘two-stage’ public goods scenario, where the secreted product catalyses the formation of an external and beneficial molecule, can be modelled by the production of a secreted catalyst *X* at rate *P* (by a population at a static density *N*), with decay rate *f*, driving the production of the beneficial molecule *Y*, formed when the catalyst molecules interact with another molecule in the environment (we assume this conversion to the beneficial molecule occurs at rate *q*, proportional to the catalyst concentration). The beneficial molecule *Y* is consumed at rate *c* and decays at rate *e*. Both *X* and *Y* are lost by mass transfer at rate *m*. These assumptions yield the following differential equations:
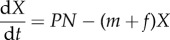
and
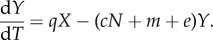

These equations yield the equilibria
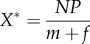
and
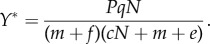

In figure 2*d*, we plot the region of parameter space where the supply of the beneficial product Y* exceeds an arbitrary threshold *y*, representing the break-even investment point (where costs equal benefits). When public goods are of the two-stage type, the threshold investment contour has positive concavity (for a mathematical proof, see Cornforth *et al*. [25]).

A two-stage public goods model predicts the environmental regime where secretion is favourable. This environmental region can be better estimated by two signals (see figure 2*c*,*d*).

The QS, DS and efficiency-sensing arguments summarized above rely on inferring environmental parameters using a single signal molecule. One possible solution to the inferential challenges of using a single molecule to discriminate distinct social (density) and physical (mass transfer) environmental regimes is to use more than one signal molecule, a common feature among generalist microbes [7]. In a recent study, we illustrated using a mix of theory and experiment that bacteria can improve discrimination of both their physical and social environment by producing and responding to multiple signals that differ in their intrinsic chemical stability [25]. While the absolute concentrations of both molecules increase with population density, variation in their ratio reveals variation in mass transfer. (At low mass transfer the more fragile molecule has time to break down in the vicinity of the sensing cells, and so the ratio shifts in favour of the more stable molecule. If the mass transfer is high, the effect of decay of the fragile molecules is masked by removal of both and the ratio does not shift in favour of the fragile molecule.) With appropriately tuned rates of signal production and signal decay, such a system enables enhanced discrimination across the two environmental parameters, density and mass transfer (box 1 and figure 2*b*). We would therefore expect that (i) signal molecules vary in their rates of chemical decay and (ii) that cells respond with combinatorial (non-additive) response rules to different signal molecule distributions. Cornforth *et al*. [25] demonstrate that *P. aeruginosa* displays diverse combinatorial gene expression responses to two signals with differential rates of decay and uses a specific AND-gate response rule to limit the expression of costly secreted factors to the most beneficial high-density, low mass transfer environments. This property of ‘combinatorial communication’ is a hallmark of human language and has recently been reported among primates [30,31]. Our work highlights that combinatorial communication has a much broader taxonomic range and is computationally achievable in single-celled organisms.

The phenomena we have described so far classes QS as a form of ‘emergent sensing’, where estimates of environmental properties arise from social interactions at the level of the collective, rather than based on enhancement of private estimates [32]. This has recently been demonstrated in schooling fish (golden shiners, *Notemigonus crysoleucas*), where collective sensing of light gradients emerges at the group level via social interactions [32]. QS functions in a similar manner as estimates of both cellular density and mass transfer arise via the social interaction of signal production. However, as well as this type of emergent or collective sensing, QS signal molecules can in principle also transmit private information among cells. A common feature of different QS systems is that they are embedded in a complex regulatory network, with both production of and responses to QS molecules being contingent upon other environmental conditions that can be directly sensed, such as stress and nutrient concentrations [33,34], leading to the production of QS molecules and the expression of QS related genes varying dramatically across different growth media [34–36]. Thus, QS molecules function both as a collective sensing mechanism and as a means of sharing private information on directly sensed environmental variables. Taken together, the role of QS as a mechanism of environmental sensing, and the influence of private information on signal production suggests that the information available via QS is rich, combinatorially integrating large numbers of environmental variables.

## 3. What does quorum-sensing control and why?

**Figure 3.**
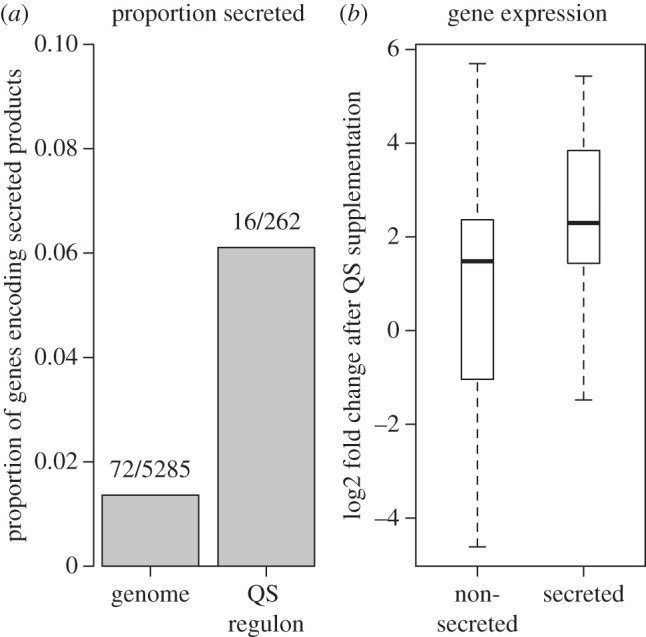
The control of secretions by QS in *P. aeruginosa*. We analysed data from a previous study where gene expression in a mutant in two AHL QS systems (PAO1 ΔlasI/rhlI) was measured with and without the supplementation of both 3-oxo-C12-HSL and C4-HSL [25]. (*a*) Genes encoding secretions are over-represented in the QS regulon (6.1%) compared to the genome as a whole (1.4%). (*b*) Genes that encode secretions are activated by QS to a higher degree than non-secretions when QS is activated by both signals.

The response to QS can be dramatic, with estimates on the proportion of the *P. aeruginosa* genome influenced by QS varying between 2 and 10% [27]. Traits under control of QS include biofilm formation [37], antibiotic production [38] and social motility mediated via biosurfactants [39]. Across this diversity of traits, one commonly reported theme is a preferential influence on secreted, extracellular traits [27,28]. Secreted products are important virulence determinants and allow generalist pathogens to colonize a wide range of environments, including new hosts [40]. Our recent microarray results [25] are consistent with preferential control of secretions by QS; the secretome (the set of genes coding for secreted proteins) represents approximately 1.4% of the PA genome, and 6.1% of the PA QS regulon (figure 3*a*), which is a significant enrichment (binomial test: percentage = 6.5%, 95% CI = 3.76% - 10.3%, *p* < 0.0001). It remains possible however that while the proportion of secreted gene products in the QS regulon is higher than in the genome at large, that the total energetic investment in secretion is no greater in the QS regulon than across the whole genome. The key question is: what proportion of the energetic cost of a response to QS is due to secretion? We found that genes encoding secretions were more highly expressed in response to QS than genes that do not encode secretions (figure 3*b*, mean expression fold change in response to QS: non-secreted = 2.03, secreted = 4.91; Welch 2 sample *t*-test, *t*_16.6_ = 2.21, *p* = 0.041). This result suggests that a disproportionate amount of the energetic cost of responding to QS is channelled into secretions. Compelling evidence that both the diversity and extent of secretions are enriched by QS comes from proteomic studies where it has been observed that 23.7% of total protein secretion is due to QS upregulation (while influencing at most 10% of the genome [27]) and that QS mutants are severely impaired in secretion [41,42]. Analyses of *Erwinia* and *Vibrio* species also implicate QS in the control of primarily secretions and secretion apparatus [43,44]. In this section, we consider the potential benefits of coupling QS regulation to the control of secreted, collective traits. Many microbes rely on active extracellular modification of their environment, secreting an array of factors to scavenge nutrients and digest extracellular macromolecules. The QS control of such traits hints that environmental manipulation via secreted enzymes is more favourable at a high local density of cells [45]. In box 2, we assess this common claim via a simple model of extracellular secreted factor dynamics (for more detailed analysis, see [25]).

In box 2, we illustrate that for simple assumptions on the extracellular dynamics of secreted factor *X*, the concentration of an extracellular beneficial product *Y* will typically be increasing with density *N*, as environmental losses (of both *X* and *Y*) become less significant as density increases. Conversely, the supply of *Y* will decrease with increasing mass transfer as both *X* and *Y* are more rapidly removed (box 2, figure 2*c,d*). Figure 2*d* illustrates the threshold above which production of secreted factor *X* is favoured given fixed costs and varying levels of density *N* and mass transfer *m*. The threshold above which production is favoured curves upward, which can make the optimal region difficult to approximate via ‘efficiency sensing’ with one signal molecule alone and can be better approximated using a combination of the two molecules (using combinatorial AND-gate signal integration, see [25]). Consistent with this prediction, we previously found an increased prevalence of combinatorial AND-gates among the QS regulatory controls of secreted factors [25]. However, it is worth noting that being able to separate the density-mass transfer plane into four quadrants can have other functional benefits as well. For instance, approximately 30 QS regulated genes are under NOR-gate control. Given the assumptions of our simple two-signal model, we would predict that they are most advantageous under conditions of low-density and high-mass transfer [25]. Detailed study of mappings between environment and gene expression are necessary to further understand these issues.

Given the expectation that population density will have a positive effect on the benefit of enzymatic secretions, we would expect to observe that larger populations grow faster, when reliant on extracellular digestion. In support of this, populations of *Myxococcus xanthus* growing on casein which must be digested extracellularly, grow at a faster rate when the population is larger [46]. A similar effect can be observed in cultures of the yeast *Saccharomyces cerevisiae* when grown on sucrose, which requires extracellular digestion via the enzyme invertase. The yeast cultures cannot establish growth on low concentrations of nutrients unless a sufficiently large innoculum is used [47]. A population can overcome this by clumping together, or flocculating, a common behaviour in naturally isolated yeast. The implication is that efficiency of growth on extracellular nutrients is enhanced by both increased population size and cell clumping. A recent study reports that QS controlled protease secretions in *P. aeruginosa* confer a larger benefit when the population is large [45]. By disabling the native QS system and experimentally reactivating it (via exogenously supplied synthetic signal molecules), the authors demonstrate that protease production, induced via supplemented QS signals, leads to a higher proportional increase in growth at high density. Similar results, supporting the conclusion of a positive density-dependent benefit of QS-controlled exoenzymes, were found in an entirely synthetic QS system [48].

The important point is that secretion behaviour in these examples is reserved via QS control for high-density environments when it will be of most benefit. We note that the functional forms of the relationships between density, exoproduct concentration and growth benefits are at present rarely measured even crudely, and yet have important implications for the evolutionary dynamics of secreted traits, as we explore in the following section.

Finally, it is important to remember that QS often exerts positive regulatory effects on non-secreted, intracellular traits. One such example is the *nuh* gene in *P. aeruginosa*, which is required for intracellular digestion of adenosine. In the case of *nuh*, the benefit derived from expressing this trait is unlikely to be affected by population density (no density dependence was observed in an experimental manipulation of adenosine concentration, [45]), rather it will be determined solely by the supply of adenosine. We have argued that QS can restrict secretions to favourable population densities given that the benefits of extracellular environmental augmentation increase with population density. Why then are intracellular traits whose benefits are independent of population density under the positive control of QS? One possibility is that selection has linked traits under the control of QS whose benefits are statistically associated with environments where population density is high. Nucleotides are likely to be in abundance when the environment contains dead cells such as during infection or competition with other bacterial colonies. The relative investment in social and asocial traits when QS is ‘on’ requires more empirical attention.

## 4. Social dilemmas and quorum sensing

In the preceding sections, we have focused on the extracellular dynamics of cell–cell signal molecules, and the secreted factors they control. We now turn to a brief discussion on the potential evolutionary dynamics of QS populations. Specifically, we focus on two dimensions of adaptation—evolutionary changes in the response to extracellular signal, specifically cooperative, extracellular responses; and evolutionary changes in the extent of signal investment.

### 4.1. Evolution of quorum-sensing-controlled cooperation

The evolutionary puzzle posed by cooperative behaviours is simple: how can cooperative (or helping) traits be maintained by selection in the face of competition with ‘cheat’ individuals that take the benefits but do not pay the costs of cooperation? In a microbial context, cooperation is widespread in the form of investment in the production of extracellular ‘public goods’; secreted factors that return benefits to neighbouring cells [49]. For every cooperative public goods trait studied, non-producer ‘cheat’ genotypes are rapidly discovered, and this raises the question of how social dilemmas are solved at a microbial scale? The QS system of *P. aeuruginosa* presents a valuable empirical model of QS social evolutionary dynamics. The tight regulation of secreted protease enzymes by QS allows QS positive populations to access nutrients in protein media while QS negative mutants do not achieve as high a density when grown alone as separate clones [50–52]. When competed against each other, a rare QS mutant, unable to respond to QS molecules with protease, can nonetheless gain access to nutrients through the production of protease by QS positive individuals [12,15]. These experiments highlight that QS controlled cooperation is costly and exploitable by non-responders or ‘cheats’, and further that the cheats reduce the virulence of infections [53,54]. A major simplification in these studies, however, is the limitation to only two fixed strategies, wild-type and non-producer ‘cheats’.

**Figure 4.**
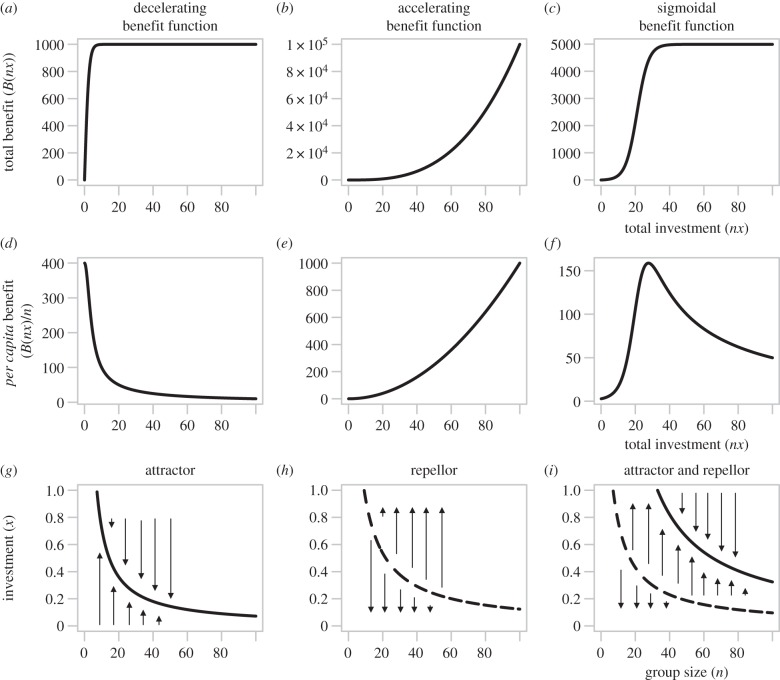
Evolutionary dynamics of cooperative secretions depend critically on nonlinearities in benefit functions. Panels (*a*–*c*) show the total group benefits of a public good for (*a*) diminishing, (*b*) accelerating and (*c*) sigmoidal returns on total group investment, with *per capita* benefits shown in (*d*–*f*) (adapted from [25]). Individual investment is set as *x* = 1 and *n* is the group size. We assume that the public good is rival (for discussion, see [57]) so that the *per capita* benefit is *B*(*nx*)/*n*, where *B*(*nx*) defines the total benefit of total group investment. The functions plotted are (*a,d,g*) *B*(*xn*) = *α*[*β* + *d*exp(κ–*bxn*)]^–1^ − *α*[*β* + *d*exp(*κ*)]^–1^, where *α* = 2000, *d* = 1, *β* = 1, *κ* = 0, *b* = 0.8; (*b*,*e*,*h*) *B*(*xn*) = *b*(*xn*)*^a^*, where *b* = 0.1 and *α* = 3; and (*c*,*f*,*i*) *B*(*xn*) = *α*[*β* + *d*exp(*κ*–*bxn*)]^–1^ − *α*[*β* + *d*exp(κ)]^–1^, where *α* = 10 000, *d* = 1, *β* = 2, *κ* = 7, *b* = 0.3. These functions are taken from [56] and are chosen for their respective shapes (diminishing, accelerating and sigmoidal). Panels (*g*–*i*) show the evolutionary dynamics of investment for each of the benefit functions. These show how selection will act on investment levels. The solid lines show evolutionary attractors, whereas dashed lines show evolutionary repellors. With decelerating benefits (*g*), there is a unique ESS, which declines with group size. With accelerating benefits (*h*) cooperation is entirely disfavoured at low group sizes, but full cooperation also becomes a stable strategy with an increasingly large basin of attraction as group size increases. With sigmoidal benefits (*i*), there can be two singular strategies, one an attractor and the other a repellor. Cooperation is entirely disfavoured at both low and high group sizes, but stable cooperation can occur at intermediate group sizes. In *g*–*i*, within group relatedness is set to *r* = 0.1, however, in all cases higher relatedness favours the evolution of cooperation [56]. The cost function used is *C*(*x*) = 5*x*. See [56] for further model details.

In order to understand the longer term evolutionary trajectories of investment in QS-controlled public goods traits, we review an existing theoretical model of group beneficial behaviours with continuously varying cooperative investment strategies using an approach termed adaptive dynamics [55,56]. This work highlights that the outcome of the social dilemma between more and less cooperative individuals is highly dependent on the shape of the relationship between the concentration of public goods and the corresponding benefit to the group. Put another way, evolutionary dynamics are contingent on the extent of additional benefit provided by each additional secreted enzyme. Despite this our current understanding of empirical cost and benefit functional forms is extremely limited. We consider three generic benefit curves (shown in figure 4*a–c*), diminishing (*a*), accelerating (*b*) and sigmoidal benefits (*c*). The benefit curves are shown both as the total benefit to the local population (*a–c*) and as a *per capita* (per cell) benefit (*d*–*f*). In the first two cases, the *per capita* benefit of each additional unit of public good provides a smaller (*d*) or greater (*e*) benefit than the previous one. In the third case, additional units of public good provide first a greater *per capita* benefit at low production levels and then decreasing benefits at higher levels (*f*). For simplicity, in all three cases the costs of production per unit secretion are constant.

In figure 4*g*–*i*, we illustrate the evolutionary dynamics of public goods production, as a function of increasing group size *n*. When benefits are diminishing there is a stable equilibrium (an evolutionarily stable strategy or ESS [58]—figure 4*g*). This means that selection will act on any small changes in public goods investment to return the trait to the equilibrium value (see arrows in figure 4*g*). When *n* = 1, the stable level of investment is high as all of the available benefits are accrued to the focal producer. However with increasing *n*, the equilibrium level of investment declines as the *per capita* share of the required collective effort declines. When benefits are accelerating, the result is an evolutionary repellor, above which full cooperation is favoured and below which cooperation collapses (figure 4*h*). This means that selection will act on any small deviations from the repellor value of investment to either (a) increase investment if above the repellor or (b) decrease the investment if below the repellor (see arrows in figure 4*h*). The level of investment at which this repellor occurs declines with *n*. At this point, we have recovered a scenario in which selection would favour positive density-dependent cooperation: increasing population size (*n*) increases the range of investment levels *x* in which full investment in cooperation is favoured. Finally, if the benefit curve is sigmoidal, this results in elements of both earlier figures; both a repellor and an upper stable level of cooperative investment (figure 4*i*). This leads to cooperative equilibria at some intermediate group sizes, while cooperation collapses if group sizes are very small. In all cases, increasing the relatedness among individuals within a group (e.g. decreasing the number of independent colony founders) increases the equilibrium level of cooperation and/or widens the range of conditions under which cooperation is favoured (see [56] for full details).

Figure 4 illustrates that accelerating (synergistic) benefits (figure 4*b,c*) can generate evolutionary repellors, leading to threshold dynamics—with selection on investment sensitive to both levels of current investment and group size [56]. In the face of fluctuating population density, a decision-making mechanism that can detect and respond to population density and constrain investment to sufficiently high population densities (QS) represents a selective advantage. Though this mechanism is advantageous whenever benefits are increasing (whether accelerating or not), it is especially advantageous when there is a repellor because QS can then protect the social trait from potentially irreversible exploitation and selection for cheats during periods at low densities [56].

The results summarized in figure 4 highlight the great sensitivity of secreted factor evolutionary trajectories (figure 4*g*–*i*) to the nature of the benefits resulting from these secreted investments (figure 4*a*–*c*). Gaining a better empirical understanding of the shapes of these benefit (and cost) functions is an important goal in this field. Specifically, more empirical work is needed to (a) map the effect of population density and mass transfer on public goods production, (b) map the relationship between public goods concentration and growth rate and (c) measure the selective benefit of density sensing mechanisms given (a) and (b). Finally, it is worth highlighting that existing theory on QS evolutionary dynamics has overlooked the parallel investment in both collective and intracellular or ‘private’ traits governed QS. It has recently been demonstrated that this can constrain the evolution of cheating strategies as a cheat then incurs a pleiotropic cost to cheating as it is impeded in its abilities to express the privately beneficial trait [16].

### 4.2. Evolution of signal investment

**Figure 5.**
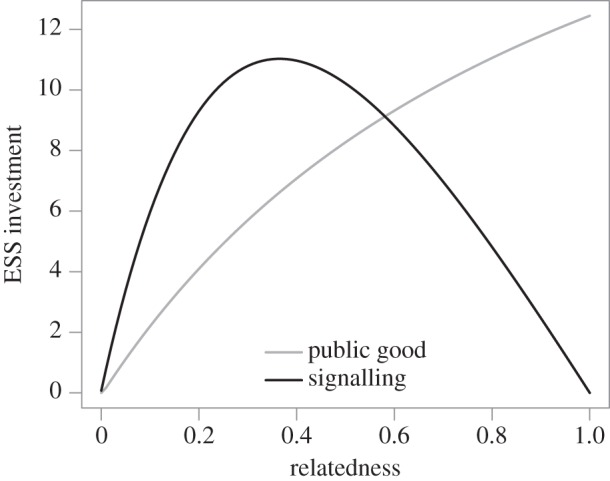
Social conflict shapes the evolution of signal production and signal response. Plotted are predictions of the model of Brown & Johnstone [59] for the evolution of investment in signalling and in cooperation. The fitness of a focal individual is given by *w*(*m, M*) = (1 − *cm*)(*p* + *nM*) − *s*, where *c* is the cost of cooperation, *p* is baseline fitness, *n* is group size (inferred from mean signal investment *S*), *s* is investment in signalling by the focal individual, *m* is the investment in cooperation by the focal individual, *M* is the average investment in cooperation in the focal individual's group and relatedness to the group *R* = d*M*/d*m*. This model leads to the prediction that, while cooperation increases monotonically with relatedness within a colony, signalling investment shows a humped relationship with relatedness. At low relatedness signalling collapses, as there is insufficient resultant cooperation. At high relatedness, minimal signalling is also favoured in order minimize the costs of regulating cooperation among identical individuals. However, at intermediate values of relatedness is at its maximum as cells attempt to ‘coerce’ cooperation from their neighbours. Parameter values are *c* = 0.04, *p* = 100 and *n* = 1000. For model details, see [59].

The level of QS molecule production is also potentially subject to social conflict, driven by the costs and benefits to individuals of producing and responding to the signal [59,60]. Experiments with *P. aeruginosa* reveal that signal production itself is costly [12], highlighting a potential individual reward for halting signal production. Conversely, in the context of a population of potential signal recipients wired to produce costly public goods in response to signal, there is also a potential reward to over-produce signal and therefore coerce neighbours into greater or earlier investments in shared public goods. Brown & Johnstone [59] developed a game-theoretical model of investments into both signal production and signal response (public goods production), and found that stable levels of investment in both signal and cooperative response can be favoured across a range of population structures. When populations exploit their environments clonally (high relatedness), investments in cooperation are high and conversely signal investment is low and constant (a ‘conspiratorial whisper’, minimizing collective signalling costs while maintaining a constant signal convention to allow inference of density). However, as within group strain mixing increases (lower relatedness) the ESS level of cooperation declines while the ESS level of signalling increases and then falls (figure 5). The initial increase in signal investment is due to the benefits of coercive strategists (high signallers, low responders) in competition with more cooperative variants (low signallers, high responders), however, as strain mixing continues to increase (lower relatedness) the diminishing levels of cooperative response ultimately make coercive investments unrewarding. The extent to which bacterial cells are selected to manipulate the behaviour of their neighbours via QS molecules has yet to be tested empirically, however, potentially coercive (high signaller) strains have been identified following experimental evolution in environments requiring collective secretions of extracellular enzymes [13].

In addition to modifying the rates of production and response to an existing signal molecule, bacteria might also adapt to conditions of social conflict by modifying the nature of the signal molecule produced, and/or their responsiveness to new and old signal variants [61]. Eldar [61] developed a theoretical model of QS evolution under conditions of genetic mixing to explore the idea that receptor genes are under selection to ignore signals and signal genes are under selection to produce variant signals that can activate the mutant receptors. The model analysis offers an account for the reported high levels of both signal and receptor diversity in several bacterial species, particularly Gram positives [62], and suggests a potentially important role of QS in bacterial kin recognition.

## 5. Quorum-sensing and antibacterial chemotherapy

The study of QS has a range of practical outputs, reflecting the centrality of QS to many aspects of bacterial life. The ability of QS to link gene expression across populations of cells has drawn attention from researchers in systems and synthetic biology [63,64], and holds the promise of novel biotechnological applications [65]. However, by far the most significant applied context is in infection biology.

Disease-causing bacteria often control a raft of virulence factors (VFs) via QS [66]. One of the most corroborated findings in the study of *P. aeruginosa* QS is that mutants in key QS components are reduced or impaired in virulence across a wide range of host species [67]. In the light of the growing crisis of antibiotic resistance, QS has therefore attracted a lot of attention as a potential route to treating bacterial infections, termed QS interference (QSI), as part of a broader initiative towards ‘anti-virulence’ therapies [68–70]. A range of different compounds with anti-virulence activity have been discovered, some of which are commercially available. The criteria to identify potential drugs are generally that the compound reduces the extent of virulence expression while not affecting growth in rich media. This property of QSI compounds and other anti-virulence drugs has prompted the claim that anti-virulence drugs will not generate selection for resistance in the same way as traditional antibiotics [68,69,71].

A number of recent studies are beginning to shed light on this ambitious claim [72–75]. The most favourable scenario is that turning off the expression of specific microbial VFs (molecular determinants of virulence in humans) presents no cost to the microbe. At first sight, it would appear unlikely that microbes deploy entirely wasteful patterns of gene expression within hosts. However, consider the example of opportunistic pathogens that live in the environment or as commensals. If selection in the non-pathogenic state is the major force maintaining VFs, then it is indeed plausible that some VFs confer no benefit to the pathogen during human infection [76]. One such case is infection with extra-intestinal pathogenic *E. coli.* The expression of extra-intestinal virulence is reliant upon VFs that normally aid in the gut commensal lifestyle, but do not contribute to growth in extra-intestinal sites [77,78], therefore turning off the expression of these factors at the extra-intestinal virulence site is unlikely to generate selection for resistance [75]. Although QS-associated VFs are key to virulence, the extent to which QS and its associated responses are adapted to hosts or the environment is not well understood. The ecology of many opportunistic pathogens would suggest that adaptations to environmental challenges could constitute a major selective force. More work is needed to measure the fitness costs and benefits endowed by VFs both in infections and in the environment or during commensal interactions with human hosts.

In the case where VFs do indeed confer benefits to pathogen growth within the host, the risks of selection for resistance are real and have been directly observed [73]. However the social, collective component of many QS-controlled VFs presents a significant impediment to the evolution of resistance, as resistance requires the restoration of a cooperative phenotype in the context of a sea of chemically induced cheats: a resistant clone may share the benefits of resistance with neighbouring cells and this could impede selection for resistance [72]. A recent experimental study points to the increased evolutionary robustness of targeting collective traits, compared with standard antibiotic treatment. Over 12 days of experimental evolution, all populations of *P. aeruginosa* exposed to a variety of different antibiotics rapidly evolved resistance. By contrast, populations exposed to a novel anti-virulence drug that extracellularly quenches a secreted VF showed no improvement in their ability to grow over the 12 days of treatment [74]. Over this short time frame at least, evolution of resistance was thwarted, despite the significant cost to bacterial growth imposed by the drug.

We believe that the ecological and evolutionary dynamics of resistance to new QSI therapeutic strategies (and other anti-virulence drugs) presents an exciting and challenging avenue of research. Key to progress in this field is the careful integration of molecular, mechanistic understanding with ecological and evolutionary dynamical modelling. With the correct combination of mechanistic design and evolution-informed stewardship, these approaches could greatly improve our ability to sustainably control pathogen-induced harm.
